# Isolation and characterization of a bacterium affiliated with the hitherto uncultured candidate phylum WOR-3 from a deep-sea hydrothermal fluid

**DOI:** 10.1128/aem.00188-25

**Published:** 2025-06-10

**Authors:** Koji Mori, Kohei Hidaka, Satoshi Tamazawa, Akira Hosoyama, Hideyuki Tamaki, Takeshi Kakegawa, Satoshi Hanada

**Affiliations:** 1 NITE Biological Resource Center (NBRC), National Institute of Technology and Evaluation (NITE), Kisarazu, Chiba, Japan; 2 Bioproduction Research Institute, National Institute of Advanced Industrial Science and Technology (AIST) 267773 , Tsukuba, Ibaraki, Japan; 3 Tohoku University 13101, Sendai, Miyagi, Japan; University of Milano-Bicocca, Milan, Italy

**Keywords:** WOR-3, EM3, Caldipriscota, Hydrothermota, Stahliibacteriota

## Abstract

A pure cultured representative, designated strain sy37, in the candidate WOR-3 lineage was obtained from a deep-sea hydrothermal fluid. This lineage was named as the candidate phylum, “*Candidatus* Caldipriscota,” “*Candidatus* Hydrothermota,” or “*Candidatus* Stahliibacteriota,” based on metagenome-assembled genomes of samples from various environments. The isolated strain was a thermophilic, microaerophilic, and chemoheterotrophic rod and obtained energy through aerobic/anaerobic respiration using oxygen and elemental sulfur. The strain could not use thiosulfate, sulfate, sulfite, fumarate, nitrate, nitrite, selenate, selenite, arsenate, ferric citrate, or ferrihydrite as an electron acceptor. Genomic annotation suggested that strain sy37 possesses a novel sulfur reduction mechanism. Among members of the WOR-3 lineage, only the group containing strain sy37 possessed the oxidative phosphorylation pathway with V-type ATPase and cytochrome *c* oxidase and may be capable of evolving to adapt to modern oxidative environments.

**IMPORTANCE:**

Genome analysis from various environments has revealed the overall diversity of microorganisms. However, there are many lineages for which culture representatives do not yet exist, and the characteristics and ecological significance of many of these microorganisms remain unclear: the WOR-3 lineage is one of these and has been found in various environments through the 16S rRNA gene analysis. In recent years, the metagenome-assembled genomes have been determined from the environments. In this study, we report on the successful isolation of a thermophilic microaerobic chemoorganoheterotroph, strain sy37, which is phylogenetically belonging to the WOR-3 lineage, from a deep-sea hydrothermal environment for the first time.

## INTRODUCTION

The accumulation of metagenome-assembled genomes (MAGs) and single-amplified genomes (SAGs) from various environments has recently revealed the existence of uncultured microbial lineages, which were classified based on their genomic information (1, 2). These phylogenetic lineages, which were once revealed only by their 16S rRNA gene sequences in samples recovered from various environments, have come to have more reality by their genome sequences. Although the physiological properties of these lineages have been inferred from annotation and environmental information obtained from the genomes, there have been few cases in which cultivated representatives have been retrieved by their information (3, 4).

The EM3 lineage is among the oldest known lineages, originally represented by the environmental clone sequences retrieved from the Octopus Spring and Obsidian Pool in Yellowstone National Park (5, 6); their MAG was first determined from a hydrothermal fluid sample from Juan de Fuca Ridge (7).

According to taxonomic rank normalization, the EM3 lineage is a class within a larger monophyletic unit comprising the candidate phylum WOR-3 and listed as a member of the WOR-3 lineage in the Genome Taxonomy Database (GTDB) (1, 8). The WOR-3 lineage was defined using MAG sequences from an estuary sediment (9), including several 16S rRNA gene sequences from marine environmental biofilms (10). Three candidate phylum names have been proposed for the WOR-3 lineage, causing some confusion (11): the phylum “*Candidatus* Pyropristinus” was proposed for the MAG sequence of “*Candidatus* Caldipriscus” sp. T1.2 from the Bechler Spring microbial filament in Yellowstone National Park in 2016 (12); the phylum “*Candidatus* Hydrothermae” was named after the representative MAG sequence of “*Candidatus* Hydrothermae pacifica” JdFR-72 retrieved from a Juan de Fuca Ridge flank subsurface fluid in 2017 (7); and the phylum “*Candidatus* Stahlbacteria” was proposed for several MAG sequences belonging to the WOR-3 lineage in the absence of representatives in 2017 (13). According to the International Code of Nomenclature of Prokaryotes, because the phylum name is formed by addition of the suffix *-ota* to the stem of the name of the designated type genus, these three candidate phylum names were corrected to “*Candidatus* Caldipriscota,” “*Candidatus* Hydrothermota,” and “*Candidatus* Stahliibacteriota,” respectively (14, 15). As a result, three phylum names remained as candidates for the WOR-3 lineage.

The WOR-3 lineage (EM3 lineage) was originally proposed based on 16S rRNA gene sequences obtained from hot springs in Yellowstone National Park (5, 6). Since then, MAG and clone 16S rRNA gene sequences have been detected in various environments, such as terrestrial hot springs (12, 16, 17), deep-sea or shallow hydrothermal vents (7, 13, 17–19), anaerobic digesters (20–24), wastewater (24), groundwaters (25, 26), a tailing pond (24), marine sediments (9, 27), and oil reservoirs and fields (28, 29). Prediction of the metabolic capability of the WOR-3 lineage based on MAG sequences suggested that they are chemoorganoheterotrophs that obtain energy for growth through aerobic respiration (12, 13, 30, 31). Although the functions of this lineage have been presumed and a certain number of the lineage is thought to be present in terrestrial hot springs, no cultured representative has been obtained.

In the present study, we report that a novel thermophilic microaerobic chemoorganoheterotroph, designated strain sy37, was isolated from a deep-sea hydrothermal environment and phylogenetically appeared to be a member of the WOR-3 lineage. In addition, we discuss the physiological and genomic characteristics of the WOR-3 lineage based on results obtained from the first cultured representative.

## RESULTS

### Morphology

**Fig 1:**
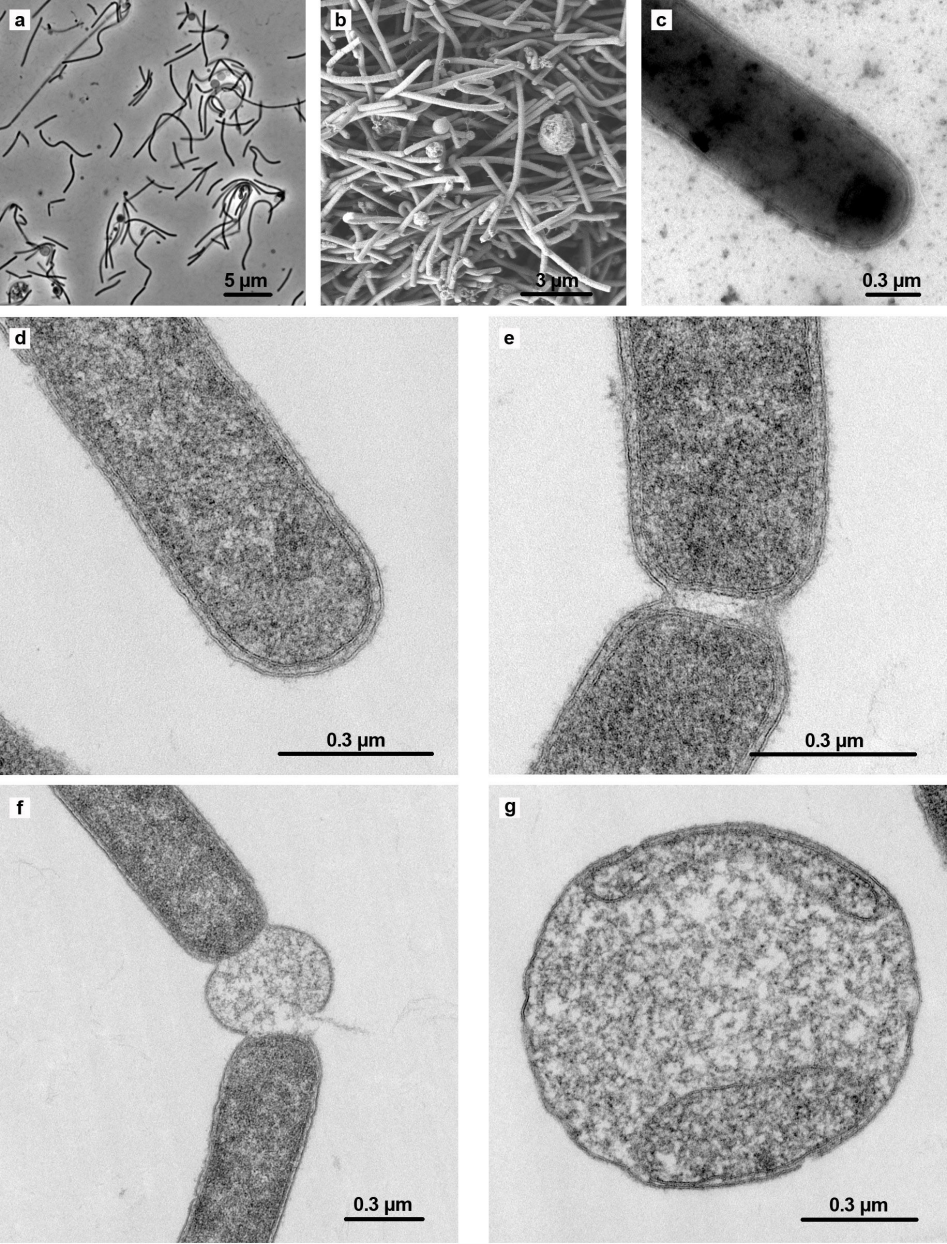
Phase contrast (**a**), scanning electron (**b**), and transmission electron (c–g) micrographs of strain sy37. Negatively stained cells are shown in (**c**). Ultrathin sections of cells are shown in (d–g).

The cells of sy37 were Gram-stain-negative, thin rods (width: 0.3 µm, length: 2–4 μm; Fig. 1a and b). The cells had a thick cell wall with inner and outer membranes (Fig. 1d and e). Pili were observed around the cells by negative staining (Fig. 1c), and motility was not observed under microscopic observation. The cells sometimes joined together to form filaments, and swollen spheres like microvesicles were often observed (Fig. 1a, b, f and g). Some spheres had membrane structures inside (Fig. 1g).

### Physiological characteristics

Strain sy37 grew under high temperature (70°C) and neutral pH conditions with yeast extract under an N_2_/CO_2_/O_2_ atmosphere. Strain sy37 could only grow under static conditions, and constantly shaking culture conditions inhibited its growth. Growth was observed at oxygen concentrations between 2% and 10%(v/v), indicating that the strain was microaerophilic, obtained energy through respiration, and did not grow through fermentation. Strain sy37 no longer grew when the cells were exposed to oxygen for extended periods under non-growth conditions (e.g., room temperature). Tests for the utilization of electron acceptors in the presence of yeast extract as a substrate revealed that sy37 could use oxygen and elemental sulfur, but not thiosulfate, sulfate, sulfite, fumarate, nitrate, nitrite, selenate, selenite, arsenate, ferric citrate, or ferrihydrite. In the presence of 5%(v/v) oxygen as an electron acceptor, growth was not observed in the medium containing various tested substrates to substitute for yeast extract and was not improved by supplementation with 0.2 g L^–1^ yeast extract. Chemoautotrophic growth of strain sy37 was not observed with H_2_/O_2_, H_2_/S^0^, H_2_/NO_3_, or S^0^/O_2_ (electron acceptor/donor) in the presence of CO_2_, and addition of 10 mM acetate did not improve growth. In the presence of 5%(v/v) oxygen at 70°C, the good growth of strain sy37 was observed (doubling time 10 h), and the maximum turbidity reached 0.15.

### Genomic feature

The genome of strain sy37 consisted of a 2,389,766 base pair circular chromosome with a G + C content of 60.6 mol%. Genome annotation with DFAST analysis revealed that the genome contained 2,083 predicted genes, including 1,107 hypothetical proteins, and 52 RNA genes consisting of four ribosomal RNAs (one 5S, two 16S, and one 23S), 47 transfer RNAs, and one transfer-messenger RNA. The homology search suggested that the 23S ribosomal RNA contained an intron encoding homing endonuclease, although this was not confirmed experimentally.

### Phylogenetic analyses

**Fig 2:**
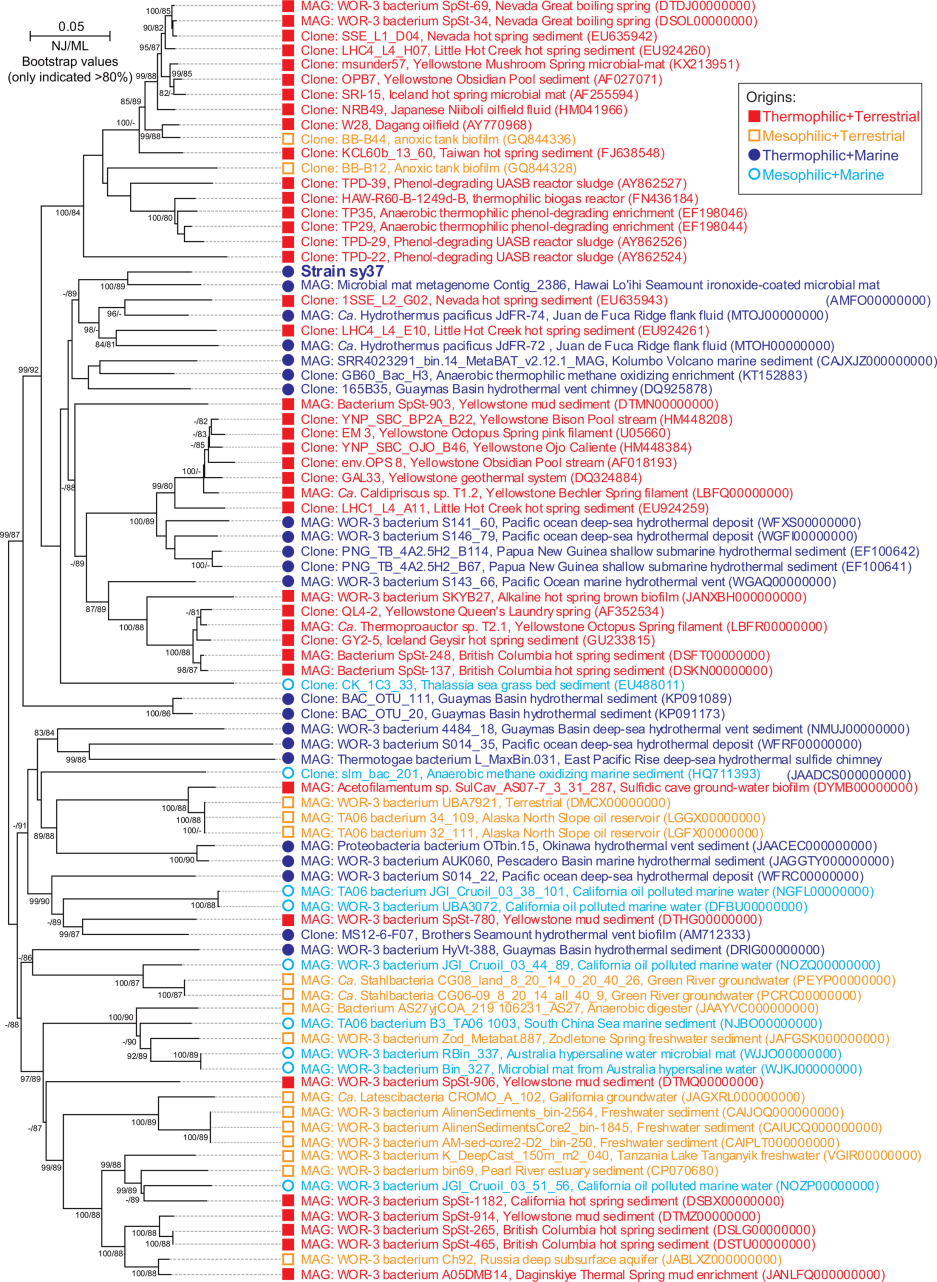
Neighbor-joining tree of strain sy37 within the WOR-3 lineage based on 16S rRNA gene sequences. The names, collection sites, and DDBJ/ENA/GenBank accession numbers are shown. The numbers are indicated at the nodes when above 80% bootstrap value was obtained (neighbor-joining method/maximum-likelihood method). Solid squares (red), open squares (orange), solid circles (dark blue), and open circles (blue) indicate origins from thermophilic terrestrial, mesophilic terrestrial, thermophilic marine, and mesophilic marine environments, respectively. Bar, 0.05 substitutions per nucleotide site.

Comparative analysis based on 16S rRNA gene sequences indicated that strain sy37 was not closely related to known cultured microorganisms and belonged to the WOR-3 lineage. Therefore, 16S rRNA gene sequences belonging to the WOR-3 lineage were collected from public databases, and the phylogenetic topology of the WOR-3 lineage, including strain sy37, was constructed using the neighbor-joining method (Fig. 2). The result obtained using the maximum-likelihood method was similar to that obtained from the neighbor-joining method (Fig. S1). Strain sy37 formed a large cluster with the WOR-3-lineage clone and MAG sequences retrieved mainly from hot environments. The closest relatives to the strain were MAG sequences of microbial mat metagenome contig_2386 (sequence similarity of 91.5%) and “*Ca*. Hydrothermae pacifica” JdFR-74 (89.1%) obtained from Juan de Fuca Ridge flank fluids (7, 32). The environmental clone and MAG sequences, GB60_Bac_H3 from the 60°C anaerobic methane oxidizing enrichment of a marine sediment (33), S143_66 and S146_79 from hydrothermal deposit in the Pacific Ocean (18), and 165B35 from a hydrothermal vent chimney of Guaymas Basin, were highly related to strain sy37 with sequence similarities of 86.5%, 86.5%, 85.3%, and 86.4%, respectively. The representatives of phylum “*Ca*. Caldipriscota,” “*Ca*. Caldipriscus” sp. T1.2, and “*Ca*. Thermoproauctor” sp. T2.1, and representative of phylum “*Ca*. Hydrothermota,” “*Ca*. Hydrothermae pacifica” JdFR-72, and JdFR-74, were included in a large cluster containing strain sy37, showing sequence similarities with strain sy37 of 84.1%, 82.6%, 83.2%, and 89.1%, respectively.

**Fig 3:**
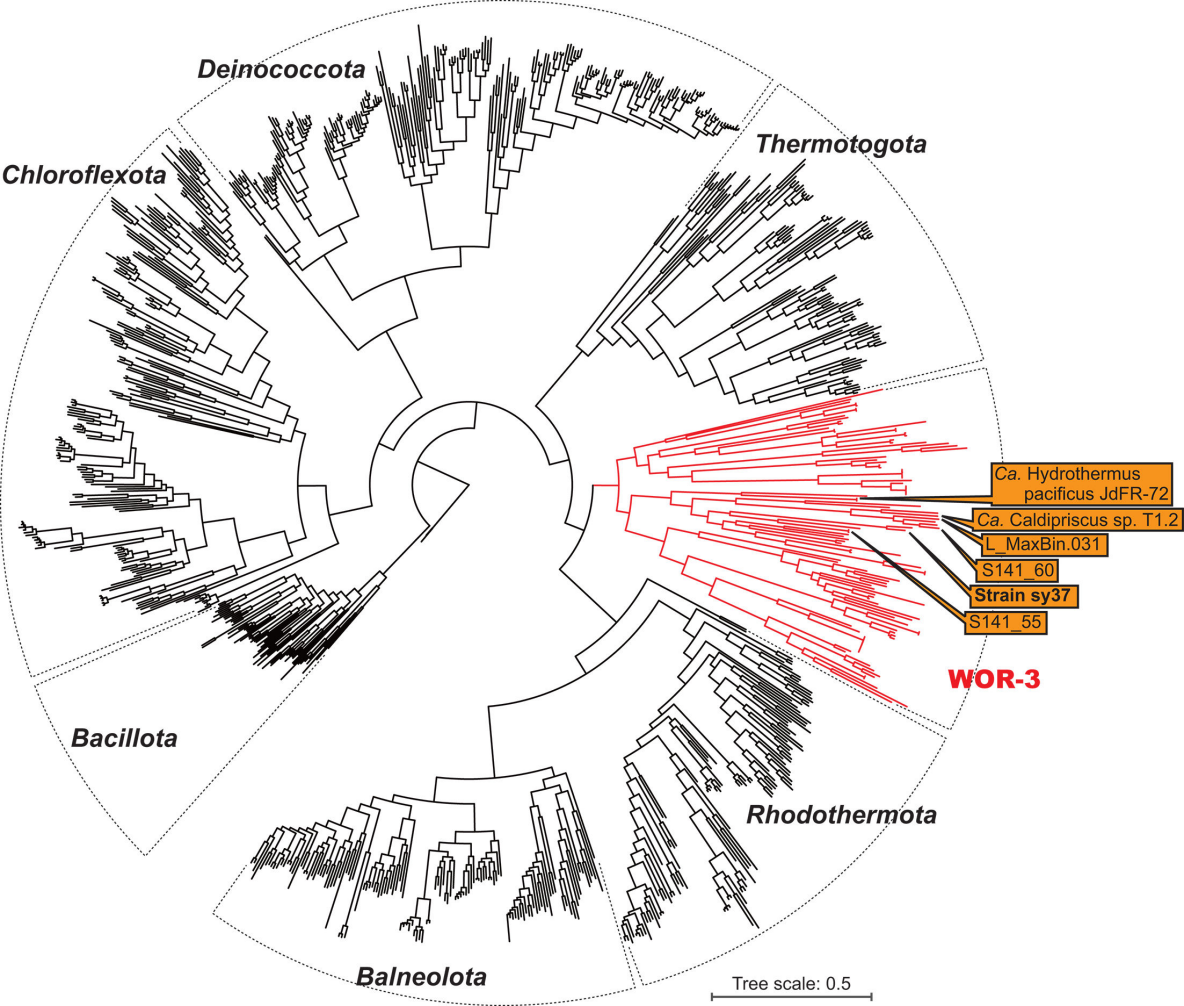
Phylogenetic tree of strain sy37 within the MAGs belonging to WOR-3 and related lineages based on conserved protein sequences inferred from GTDB-Tk. The accession numbers used for the analysis are shown in Table S1. Bar, 0.5 substitutions per amino acid site.

Genome-based phylogeny analysis was conducted with seven phyla, including the WOR-3 lineage (Table S1), indicating that the WOR-3 lineage was a monophyletic lineage at the phylum level, and strain sy37 was located within the MAGs consisting of the WOR-3 lineage (Fig. 3).

### Metabolic pathway inference of strain sy37 based on the genome

Metabolic reconstruction of strain sy37 was conducted using its genomic annotation. Strain sy37 contained all genes necessary for glycolysis and gluconeogenesis, as well as nearly all genes for the complete oxidative tricarboxylic acid (TCA) cycle. Representative CO_2_ fixation pathways were not present, and there was no key enzyme for the reductive TCA cycle. By contrast, strain sy37 may possess acetyl-CoA synthetase (SYKM_02060), which converts acetate to acetyl-CoA forms.

Regarding energy conservation, sy37 had complete NADH-quinone oxidoreductase and nearly complete cytochrome *c* oxidase. In addition, the genes of bacterial and archaeal V (vacuolar)-type ATPase were nearly completely present in the genome. Therefore, the genomic annotation also suggested that strain sy37 could utilize oxygen for respiration and conduct oxidative phosphorylation. Although nitrate reduction was not observed in the electron acceptor test, strain sy37 contained alpha- and beta-subunits of nitrate reductase for dissimilatory nitrate reduction (SYKM_13860 and SYKM_13870) but not nitrite reductase. Although genes, such as *psr* and *dsr*, were not present for representative sulfur/sulfate reduction, strain sy37 may contain a sulfide dehydrogenase-like protein (SYKM_11010, 750 aa), SudA, which *Pyrococcus furiosus* uses for sulfur reduction (34, 35). The SudA-like protein of strain sy37 was 40.2% sequence similar to that of *P. furiosus* and was longer than that of *P. furiosus* by 276 amino acids.

Complete genes for biosynthesis of DAP-type peptidoglycan and some genes for lipopolysaccharide biosynthesis for the major component of outer membrane of Gram-negative bacteria were present in the genome of strain sy37. There were few ATP-binding cassette transporters. Strain sy37 contained Cu-Zn and Fe-Mn superoxide dismutases (SYKM_02940 and SYKM_17780, respectively) but not catalase.

### Comparison of metabolic pathways among MAGs of the WOR-3 lineage based on the genome

**Fig 4:**
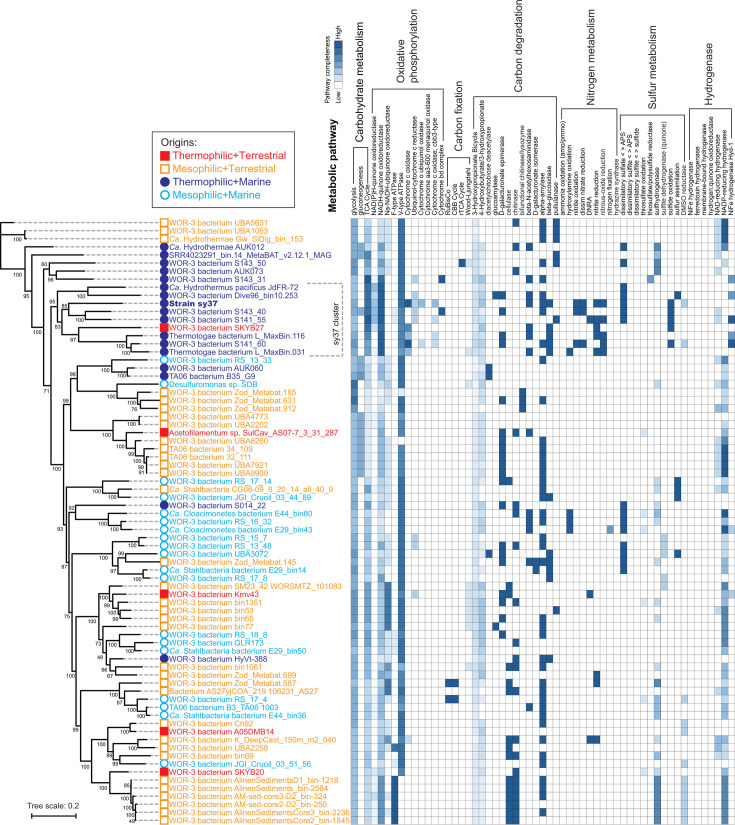
Phylogenetic tree based on conserved protein sequences among strain sy37 and the MAGs belonging to the WOR-3 lineage, and the heat map of their metabolic pathway completeness inferred from PhyoPhlAn and KEGG-Decoder, respectively. This figure is an excerpt of Fig. S2. The accession numbers used for the analysis are shown in Table S1. Bootstrap probabilities are indicated at branching points. Solid squares (red), open squares (orange), solid circles (dark blue), and open circles (blue) indicate origins from thermophilic terrestrial, mesophilic terrestrial, thermophilic marine, and mesophilic marine environments, respectively. In the map, dark blue colors represent complete or highly complete pathways, and lighter colors do non- or highly incomplete pathways. Bar, 0.2 substitutions per amino acid site.

By using the genome of sy37 and 74 MAGs belonging to the WOR-3 lineage (>90% completeness and <10% contamination values by CheckM; Table S1), the phylogenetic tree was reconstructed, and the completeness of their metabolic pathways was estimated via KEGG-Decoder analysis. The results are shown in Fig. S2; some excerpts of metabolic pathways are shown in Fig. 4. Metabolic pathway prediction showed that the WOR-3 lineage is a heterotroph that obtains energy through oxidative phosphorylation: it tends to possess NADH-quinone oxidoreductase and V-type ATPase but not CO_2_ fixation pathways. We manually confirmed that strain sy37 contained all genes for glycolysis and gluconeogenesis, but the genes showed low completeness in KEGG-Decoder analysis (Fig. 4); thus, the results of KEGG-Decoder may be underestimated. MAGs retrieved from marine thermophilic environments phylogenetically clustered together in close proximity, with strain sy37 included in the cluster (shown as the sy37 cluster in Fig. 4). Although the MAG of “*Ca*. Caldipriscus” sp. T1.2 was not included in this analysis, the phylogenetic relationship shown in Fig. 3 suggests that “*Ca*. Caldipriscus” sp. T1.2 is included in this cluster. Compared with the others, strain sy37 and the MAGs in this cluster tended to possess respiratory-related metabolic pathways such as cytochrome *c* oxidase and nitrogen reduction/oxidation metabolism. By contrast, the WOR-3 lineage, except for this cluster, tended to have a NADP-reducing hydrogenase.

## DISCUSSION

A novel thermophilic bacterium was isolated from a thermal fluid collected from a deep-sea hydrothermal field in Suiyo Seamount. Phylogenetic analyses based on the 16S rRNA gene and genome revealed that the isolate, named strain sy37, was the first cultivated representative of the uncultured lineage WOR-3. Using some annotated information from the MAGs, the metabolic functions of the WOR-3 lineage were predicted. Dombrowski et al*.* suggested that the MAGs of the phylum “*Ca*. Stahlbacteria” from a hydrothermal sediment utilized fermentative metabolism (13). Colman et al. predicted that “*Ca*. Caldipriscus” sp. T1.2 and “*Ca*. Thermoproacutor” sp. T2.1 in the lineage retrieved from microbial streamers in Yellowstone hot springs were chemoorganoheterotrophs that obtained energy for growth using aerobic respiration and oxidative phosphorylation via V-type ATPase (12). In addition, some MAGs belonging to “*Ca*. Caldipriscus” from hot spring sediments may also be aerobic chemoorganoheterotrophs (30, 31). These predictions were accurate to some extent; however, because these functional estimates were based on well-known metabolic information, the ability to predict uncultured microbial communities at the phylum level is limited. Possibly, the isolate does not represent the typical characteristics of the WOR-3 lineage. However, according to the analysis of the first cultivated strain, the strain is microaerobic, and shaking conditions have a negative effect on its growth. These factors may contribute to the difficulty of obtaining isolates in this lineage.

The morphology of sy37 was unique, with long, thin cells that had thick Gram-negative-type cell walls (Fig. 1d) and swollen spheres like microvesicles, which occasionally had membrane structures (Fig. 1a, b, f and g). The primary function of the cell wall is to protect the cell from the environment. However, the rationale for strain sy37’s heightened sensitivity to shaking conditions, in contrast to other Gram-negative bacteria, remained unclear regarding its cellular architecture. Among the long rods, various swollen spheres were observed alone, which were sometimes attached to rods (Fig. 1a and b). Some of these spheres had membrane structures (Fig. 1g), but their role is unknown.

Phenotypic growth tests indicated that strain sy37 was a chemoorganoheterotroph that obtained energy through aerobic or anaerobic respiration. Genomic annotation regarding central carbon metabolism supported the absence of a well-known CO_2_ fixation pathway, although gluconeogenesis from acetic acid may occur. Regarding energy conservation, both culture experiments and genomic information demonstrated that strain sy37 used O_2_ as an electron acceptor. By contrast, strain sy37 grew well in the presence of elemental sulfur as an electron acceptor, but well-known key genes for sulfur reduction, such as *psr* and *dsr,* were not present. Instead, strain sy37 possessed the gene of sulfide dehydrogenase-like protein, SudA (SYKM_11010). The sulfide dehydrogenase of *P. furiosus* is a heterodimer composed of SudA and SudB, and reduces polysulfide to sulfide *in vitro* (34). SudA of *P. furiosus* is highly homologous to KOD1-GltA of *Pyrococcus kodakaraensis*, which was identified as glutamate synthase but does not have glutamate synthase activity (36). In addition, to date, there is no evidence that the sulfide dehydrogenase of *P. furiosus* is involved in sulfur reduction *in vitro* or in its electron transfer system (35, 37, 38). However, a gene highly homologous to SudA of *P. furiosus* is encoded in *Staphylothermus marinus* and *Caldisericum exile*, which require sulfur compounds for growth but whose sulfur reductase is unknown (39, 40). Further investigations are required to determine the sulfur reduction mechanism of strain sy37, but it likely obtains energy through a previously unknown sulfur reduction mechanism. The cultured representative strain obtained in this study is very important for understanding the unknown sulfur reduction mechanism.

Regarding energy conservation in the WOR-3 lineage, Colman et al. reported that “*Ca*. Caldipriscus” sp. T1.2 and “*Ca*. Thermoproacutor” sp. T2.1 first utilizes V-type ATPase for oxidative phosphorylation (12). Our analysis revealed that strain sy37 possessed a V-type ATPase, which is likely a common feature in many members of the WOR-3 lineage (Fig. 4). Usage of V-type ATPase for the H^+^ pump is unique in prokaryotes but has also been reported in *Thermus thermophilus*, “*Ca*. Acetothermum autotrophicum,” *Caldisericum exile*, and others (41–43). Regarding the carbon metabolism in the WOR-3 lineage, the metabolic pathways of glycolysis and the TCA cycle were conserved, although there were varying degrees of completeness (Fig. 4). Therefore, it is of interest that only the sy37 cluster related to strain sy37 and “*Ca*. Caldipriscus” sp. T1.2, retrieved from hot marine/terrestrial environments, in the WOR-3 lineage showed high completeness of TCA cycle metabolism and possessed cytochrome c oxidases (Fig. 4) (12). Based on these results, the cluster may have evolved to be the most adapted to modern oxidative environments among the WOR-3 lineage. By contrast, the general energy acquisition mechanism associated with V-type ATPase in the WOR-3 lineage remains unclear. The sulfur reduction respiratory suggested as a possibility in strain sy37 may be one such candidate for the mechanism. Further analyses using metabolic estimation and genomic data decoding of isolates and MAGs are needed to characterize the WOR-3 lineage.

## MATERIALS AND METHODS

### Sample collection, enrichment, and isolation

The hydrothermal fluid for enrichment and isolation was collected from a deep-sea hydrothermal vent chimney in Suiyo Seamount, the Izu-Bonin Arc, in the western Pacific Ocean by DSV *Shinkai6500* during the YK11-06 scientific cruise aboard the R/V *Yokosuka* (JAMSTEC, Yokosuka, Kanagawa, Japan) in August 2012. The region has a submarine caldera with numerous hydrothermal vents at a depth of 1,390 m (44). The *in situ* measured temperature of fluid was approximately 70°C. The fluid was collected in a heat-resistant water bag and immediately used for isolation on board.

The medium under a N_2_/CO_2_/O_2_ (78:20:2 [v/v], 150 kPa) atmosphere was added to a vial sealed with a butyl rubber stopper and aluminum cap for enrichment and isolation. The medium was comprised of (L^−1^) 0.6 g KH_2_PO_4_, 0.1 g K_2_HPO_4_, 0.75 g MgCl_2_·6H_2_O, 0.15 g CaCl_2_·2H_2_O, 0.3 g NH_4_Cl, 30 g NaCl, 0.3 g Na_2_SO_4_, 1 g Bacto Yeast Extract (Difco, Detroit, MI, USA), 2 mL trace element solution (45), 2 mL vitamin solution (45), and 1 g Na_2_CO_3_. After mixing these components, except for KH_2_PO_4_, K_2_HPO_4_, vitamin solution, and Na_2_CO_3_, the medium was autoclaved under a N_2_/CO_2_/O_2_ atmosphere. KH_2_PO_4_ and K_2_HPO_4_ solutions were autoclaved separately and then added to the medium. The vitamin and Na_2_CO_3_ solutions were sterilized by filtration. After 1 week of cultivation at 70°C, bacterial growth was confirmed. Following the enrichment, tiny white colonies were allowed to form for 4 days on medium solidified with 0.6% (w/v) gellan gum in vials. After a second purification step using the same solid medium, a pure culture of strain sy37 (NBRC 116057) was obtained.

Based on subsequent analysis of the cultivation conditions, unless specifically described, the modified medium was routinely used with the Bacto Yeast Extract concentration changed to 2 g L^−1^ and atmosphere changed to N_2_/CO_2_/O_2_ (75:20:5 [v/v], 150 kPa); and the strain was grown under static conditions at 70°C.

### Microscopic observation

Cell morphology was routinely observed using phase-contrast microscopy (model AX-70; Olympus, Tokyo, Japan). The cell structure was observed using scanning electron microscopy (S-4500; Hitachi, Japan) and transmission electron microscopy (H-7600; Hitachi, Japan) (46, 47). Briefly, for scanning electron microscopy observation, the cells were fixed with 2% glutaraldehyde, postfixed with 1% osmium tetroxide, and dehydrated through a graded ethanol series followed by 3-methylbutyl acetate. For transmission electron microscopy observation, the cells were fixed with 2.5% glutaraldehyde and then postfixed with 1% osmium tetroxide. The fixed cells were suspended in 1% aqueous uranyl acetate.

### Physiological characterization

Optical density (A660) was measured with a spectrophotometer (model U-2800; Hitachi, Japan). The concentrations of sulfate, thiosulfate, and nitrate were assessed using high-performance liquid chromatography (model 2695 with conductivity detector model 432 and an IC-Pac Anion column; Waters, Milford, MA, USA) (48).

The following substrates were examined as the sole energy and carbon sources: 10 mM D-glucose, 10 mM D-fructose, 10 mM D-mannose, 10 mM D-galactose, 10 mM D-melibiose, 10 mM maltose, 10 mM lactose, 10 mM D-trehalose, 10 mM sucrose, 10 mM D-cellobiose, 10 mM D-raffinose, 10 mM D-arabinose, 10 mM L-rhamnose, 10 mM D-xylose, 10 mM D-ribose, 10 mM ribitol, 10 mM D-mannitol, 10 mM D-sorbitol, 20 mM glycerol, 20 mM citrate, 20 mM pyruvate, 20 mM succinate, 20 mM malate, 20 mM L-glutamate, 20 mM butyrate, 20 mM lactate, 20 mM propionate, 10 mM acetate, 5 g L^–1^ starch, 2 g L^–1^ yeast extract, 2 g L^–1^ polypeptone, and 2 g L^–1^ casamino acids. The substrate utilization test was also performed in the presence of 0.2 g L^–1^ yeast extract. The possibility of chemoautotrophic growth was examined using a combination of the following materials under N_2_/CO_2_/O_2_ (75:20:5 [v/v], 150 kPa), N_2_/CO_2_ (80:20 [v/v], 150 kPa), H_2_/CO_2_/O_2_ (75:20:5 [v/v], 150 kPa), or H_2_/CO_2_ (80:20 [v/v], 150 kPa) conditions: H_2_, O_2_, CO_2_, 0.5%(w/v) elemental sulfur, 10 mM nitrate and 10 mM acetate.

Utilization of the following electron acceptors was evaluated in the presence of 2 g L^–1^ yeast extract as the substrate: 10 mM thiosulfate, 10 mM sulfate, 2 and 5 mM sulfite, 5 g L^–1^ elemental sulfur, 10 mM fumarate, 10 mM nitrate, 2 and 5 mM nitrite, 5 mM selenate, 5 mM selenite, 5 mM arsenate, 5 mM ferric citrate, 0.1%(w/v) ferrihydrite (49), and 5%(v/v) O_2_.

### Genome sequencing

Genomic DNA was extracted using an EZ1 Tissue kit according to the manufacturer’s instructions (Qiagen, Hilden, Germany). Whole-genome shotgun sequencing was performed using the 454 GS FLX-Titanium system (Roche, Basel, Switzerland) and MiSeq (Illumina, San Diego, CA, USA). Reads were assembled using Newbler assembler version 2.8 (Roche), and then four contigs were obtained. Primer walking on gap-spanning PCR products from genomic DNA closed the gaps between the assembled sequences: PCR primers for gap1, 5′-GGGATCGCAGGGCCCATGAA-3′ (positions 370101–370120) and 5′-TCCCTCCAGCGACAGCCGTA-3′ (positions 371245–371226); PCR primers for gap2, 5′-CAATGGCGGAAATGGGGGAC-3′ (positions 1180078–1180097) and 5′-GACCAGCGTTCGCCTGGACA-3′ (positions 1181661–1181642); PCR primers for gap3, 5′-CTCCCGGTTCAGGGGGTTCC-3′ (positions 1606581–1606660) and 5′-ACACCTGCCCGTGCTTCCAG-3′ (positions 1607257–1607238); PCR primers for gap4, 5′-CCCGGATCACGGGCCCTTTC-3′ (positions 2137474–2137493) and 5′-GAGAGGTTCCGGATGGGCCG-3′ (positions 2138959–2138940). The determined genome sequence was automatically annotated using DFAST ver.1.4.0, DDBJ Fast Annotation and Submission Tool (50, 51).

### Phylogenetic and genomic analyses

16S rRNA gene sequence was compared with the BLAST program from the National Center for Biotechnology Information (https://www.ncbi.nlm.nih.gov/) and by using the ARB program (52) with SILVA database version 138 (53). The 16S rRNA gene sequences related to the WOR-3 lineage were obtained from the National Center for Biotechnology Information and SILVA. Phylogenetic trees based on 16S rRNA gene sequences were reconstructed by the neighbor-joining method using the CLUSTAL X (54, 55), the maximum-likelihood method by using the MEGA X ver.10.1.8 (56) after alignment using CLUSTAL X.

Based on genome sequences, phylogenetic trees of strain sy37 and several taxonomic lineages (p__Thermotogota, o__Syntrophomonadales, o__Thermoflexales, c__Rhodothermia, c__Chloroflexia, p__Deinococcota) were reconstructed using GTDB-Tk ver.0.3.2 gtdbtk de_novo_wf (57, 58). In addition, for phylogenetic analysis of strain sy37 and the WOR-3 lineage, PhyloPhlAn ver.3.0.67 was used to build a maximum-likelihood tree (59). Genome sequence files of WOR-3 genomes were downloaded from NCBI on July 16, 2023, using the NCBI taxonomy database (bac120_taxonomy_r214 https://gtdb.ecogenomic.org/downloads) and genome assembly reports (https://ftp.ncbi.nlm.nih.gov/genomes/ASSEMBLY_REPORTS/assembly_summary_genbank.txt). Among them, the sequence qualities were estimated using CheckM ver.1.0.11 (60), and high-quality sequences with contamination less than 10% and completeness over 90% were only used for the analysis of PhyloPhlAn (Table S1). iTOL was used to visualize the phylogenetic trees (61).

For proteomic comparison, KEGG-Decoder (https://github.com/bjtully/BioData/tree/master/KEGGDecoder) was used (62). Genome sequence of strain sy37 was annotated by DFAST ver.1.4.0 (50), whereas gene prediction of MAGs in the WOR-3 lineage was conducted by Prodigal ver.2.6.3 (63). Annotation was conducted using eggNOG-mapper ver.2.1.10 (64), and assigned Kyoto Encyclopedia of Genes and Genomes (KEGG) orthology numbers of all strains were used to determine the completeness of metabolic pathways using KEGG-Decoder.

## Data Availability

Strain sy37 was deposited in the NITE Biologicla Resource Center as NBRC 116057. The genome sequence of strain sy37 was deposited in DDBJ/ENA/GenBank with the accession number AP038914 under the BioProject accession number PRJDB19856 and BioSample accession number SAMD00856355.

## SUPPLEMENTAL MATERIAL

Supplemental material aem.00188-25-s0001.pdf Table S1; Fig. S1 and S2.

ASM does not own the copyrights to Supplemental Material that may be linked to, or accessed through, an article. The authors have granted ASM a non-exclusive, world-wide license to publish the Supplemental Material files. Please contact the corresponding author directly for reuse.
