# Supplementary material for: Isolation and characterization of a bacterium affiliated with the hitherto uncultured candidate phylum WOR-3 from a deep-sea hydrothermal fluid
Source: Appl Environ Microbiol. 2025 Jun 10;91(7):e00188-25. doi: 10.1128/aem.00188-25 (PMC12285271; doi:10.1128/aem.00188-25)
Supplement: Supplemental material — Table S1; Fig. S1 and S2. [file aem.00188-25-s0001.pdf]

Supplemental information for

Isolation and characterization of a bacterium affiliated with the hitherto uncultured candidate phylum WOR-3 from a deep-sea hydrothermal fluid

Koji Mori[1], Kohei Hidaka[1], Satoshi Tamazawa[1], Akira Hosoyama[1], Hideyuki Tamaki[2], Takeshi Kakegawa[3] and Satoshi Hanada[2]

[1]NITE Biological Resource Center (NBRC), National Institute of Technology and Evaluation (NITE), 2-5-8 Kazusakamatari, Kisarazu, Chiba 292-0818, Japan

[2]Bioproduction Research Institute, National Institute of Advanced Industrial Science and Technology (AIST), 1-1-1 Higashi, Tsukuba, Ibaraki 305-8566, Japan

[3]Tohoku University, Sendai, Miyagi 980-8578, Japan

Corresponding author:

Koji Mori

Email address: mori-koji@nite.go.jp

Table S1 Genome sequences and information used for analysis in Figures 3 and 5.

Names	Isolation sources	Accession No.	Assemble No.	Comple- -teness by CheckM	Contam -ination by CheckM	Analysis for Fig. 3	Analysis for Fig. 5
Strain sy37	Suiyo Seamount deep-sea hydrothermal fluid	AP038914		98.31	0.00	1	1
Acetofilamentum sp. SulCav_AS07-7_3_31_287	Sulfidic cave ground-water biofilm	DYMB00000000	GCA_020721805.1	96.70	0.00	1	1
Bacterium 1244-C1-H3-B19 C1H3_C100	Pacific methane hydrate marine sediment	WJON00000000	GCA_009619125.1	60.60	0.00	1	
Bacterium AS27yjCOA_219 106231_AS27	Anaerobic digester	JAAYVC000000000	GCA_012517375.1	96.15	1.10	1	1
Bacterium B12_G15	Guaymas Basin deep-sea hydrothermal vent sediment	QNDZ00000000	GCA_003646055.1	79.81	1.75	1	
Bacterium B54_G16	Guaymas Basin deep-sea hydrothermal vent sediment	QNDX00000000	GCA_003644995.1	68.13	2.36	1	
Ca. Caldipriscus sp. T1.2	Yellowstone Bechler Spring filament	LBFQ00000000	GCA_000980735.1	58.59	1.42	1	
Ca. Cloacimonetes bacterium E29_bin43	Atlantic Ocean deep sea petroleum seepage sediments	SOIS00000000	GCA_004375965.1	95.60	2.52	1	1
Ca. Cloacimonetes bacterium E44_bin50	Atlantic Ocean deep-sea petroleum seepagesediments	SOKD00000000	GCA_004376305.1	80.03	5.24	1	
Ca. Cloacimonetes bacterium E44_bin80	Atlantic Ocean deep sea petroleum seepage sediments	SOKM00000000	GCA_004375785.1	94.51	0.00	1	1
Ca. Hydrothermae AUK012	Pescadero Basin marine hydrothermal sediment	JAGGSC000000000	GCA_021159285.1	91.58	2.30	1	1
Ca. Hydrothermae B15_G9	Guaymas Basin deep-sea hydrothermal vent sediments	QNDP00000000	GCA_003645975.1	75.32	1.20	1	
Ca. Hydrothermae B29_G16	Guaymas Basin deep-sea hydrothermal vent sediments	QNDO00000000	GCA_003644895.1	88.21	1.47	1	
Ca. Hydrothermae Gw_SlDig_bin_153	Wastewater	JAGNLS000000000	GCA_017999445.1	96.61	0.00	1	1
Ca. Hydrothermae JdFR-71	Juan de Fuca Ridge basaltic crustal fluid	MTOG00000000	GCA_002010635.1	81.36	0.00	1	
Ca. Hydrothermus pacificus JdFR-72	Juan de Fuca Ridge flank fluid	MTOH00000000	GCA_002011615.1	91.53	0.00	1	1
Ca. Latescibacteria CROMO_A_102	Galifornia groundwater	JAGXRL000000000	GCA_018335915.1	89.26	2.50	1	
Ca. Stahlbacteria	Green River groundwater	PCRC00000000	GCA_002771845.1	95.60	2.75	1	1
Ca. Stahlbacteria	Green River groundwater	PEYP00000000	GCA_002774315.1	67.60	1.12	1	
Ca. Stahlbacteria bacterium E29_bin14	Atlantic Ocean deep sea petroleum seepage sediments	SOIB00000000	GCA_004377355.1	95.14	0.00	1	1
Ca. Stahlbacteria bacterium E29_bin50	Atlantic Ocean deep sea petroleum seepage sediments	SOIY00000000	GCA_004376785.1	94.80	2.20	1	1
Ca. Stahlbacteria bacterium E44_bin36	Atlantic Ocean deep sea petroleum seepage sediments	SOJW00000000	GCA_004375895.1	94.61	0.00	1	1
Desulfuromonas sp. SDB	San Diego Bay contaminated sediments	LKUE00000000	GCA_001412365.1	95.24	2.20	1	1
Proteobacteria bacterium OTbin.15	Okinawa hydrothermal vent sediment	JAACEC000000000	GCA_011682715.1	77.86	0.00	1	
SRR4023291_bin.14_MetaBAT_v2.12.1_MAG	Kolumbo Volcano marine sediment	CAJXJZ010000000	GCA_913055745.1	93.41	0.00	1	1
TA06 bacterium 32_111	Alaska North Slope oil reservoir	LGFX00000000	GCA_001508335.1	94.51	0.00	1	1
TA06 bacterium 34_109	Alaska North Slope oil reservoir	LGGX00000000	GCA_001508575.1	93.02	3.30	1	1
TA06 bacterium B3_TA06 1003	South China Sea marine sediment	NJBO00000000	GCA_005223075.1	97.80	0.00	1	1
TA06 bacterium B35_G9	Guaymas Basin deep-sea hydrothermal vent sediment	QNBC00000000	GCA_003641665.1	94.06	0.00	1	1
TA06 bacterium DG_78 15855	North Carolina methane-rich estuary sediment	LJNI00000000	GCA_001303225.1	75.60	1.19	1	
TA06 bacterium JGI_Cruoil_03_38_101	California oil polluted marine water	NGFL00000000	GCA_002215665.1	89.64	0.40	1	
Thermotogae bacterium L_MaxBin.031	East Pacific Rise deep-sea hydrothermal sulfide chimney	JAADCS000000000	GCA_013154295.1	92.24	5.63	1	1
Thermotogae bacterium L_MaxBin.116	East Pacific Rise deep-sea hydrothermal sulfide chimney	JAADDJ000000000	GCA_013153965.1	90.52	0.00	1	1
WOR-3 bacterium 4484_100	Guaymas Basin deep-sea hydrothermal vent sediment	MUKB00000000	GCA_002049785.1	58.91	1.10	1	
WOR-3 bacterium 4484_18	Guaymas Basin deep-sea hydrothermal vent sediment	NMUJ00000000	GCA_002256535.1	53.30	0.55	1	
WOR-3 bacterium A05DMB14	Daginskiye Thermal Spring mud enrichment	JANLFQ000000000	GCA_024653355.1	95.44	0.00	1	1
WOR-3 bacterium AlinenSediments_bin-2564	Freshwater sediment	CAIJOQ000000000	GCA_903822355.1	93.38	2.25	1	1
WOR-3 bacterium AlinenSedimentsCore2_bin-1845	Freshwater sediment	CAIUCQ000000000	GCA_903897735.1	93.38	2.25	1	1
WOR-3 bacterium AlinenSedimentsCore3_bin-2236	Freshwater sediment	CAIWXC000000000	GCA_903916525.1	93.19	2.25	1	1

WOR-3 bacterium AlinenSedimentsD0_bin-1181	Freshwater sediment	CAIVTL000000000	GCA_903908785.1	84.75	2.85	1	
WOR-3 bacterium AlinenSedimentsD1_bin-1218	Freshwater sediment	CAISFH000000000	GCA_903884565.1	93.38	1.12	1	1
WOR-3 bacterium AlinenSedimentsD2_bin-0689	Freshwater sediment	CAISUA000000000	GCA_903888505.1	81.43	3.37	1	
WOR-3 bacterium AM-sed-core2-D2_bin-250	Freshwater sediment	CAIPLT000000000	GCA_903865935.1	93.38	1.19	1	1
WOR-3 bacterium AM-sed-core3-D2_bin-324	Freshwater sediment	CAITFA000000000	GCA_903891615.1	93.19	1.23	1	1
WOR-3 bacterium AUK060	Pescadero Basin marine hydrothermal sediment	JAGGTY000000000	GCA_021158765.1	90.11	1.71	1	1
WOR-3 bacterium AUK073	Pescadero Basin marine hydrothermal sediment	JAGGUL010000000	GCA_021158525.1	91.21	0.06	1	1
WOR-3 bacterium B36_G15	Guaymas Basin deep-sea hydrothermal vent sediment	QNBE00000000	GCA_003645615.1	89.01	1.75	1	
WOR-3 bacterium Bin_327	Microbial mat from Australia hypersaline water	WJKJ00000000	GCA_014729155.1	76.37	0.00	1	
WOR-3 bacterium bin1061	Pearl River estuary sediment	CP070834	GCA_020341755.1	90.56	0.00	1	1
WOR-3 bacterium bin1351	Pearl River estuary sediment	CP070802	GCA_020343595.1	94.51	1.10	1	1
WOR-3 bacterium bin53	Pearl River estuary sediment	CP070705	GCA_020349965.1	95.60	0.10	1	1
WOR-3 bacterium bin65	Pearl River estuary sediment	CP070686	GCA_020353055.1	96.70	1.10	1	1
WOR-3 bacterium bin69	Pearl River estuary sediment	CP070680	GCA_020352555.1	90.05	0.00	1	1
WOR-3 bacterium bin77	Pearl River estuary sediment	CP070664	GCA_020355325.1	94.51	0.00	1	1
WOR-3 bacterium Ch92	Russia deep subsurface aquifer	JABLXZ000000000	GCA_013177935.1	95.51	0.00	1	1
WOR-3 bacterium Dive96_bin10.253	Indian Ridge Longqi hydrothermal vent active black smoker	JAJRUZ000000000	GCA_024277525.1	93.22	0.00	1	1
WOR-3 bacterium GLR173	Glendhu Ridge methane seep	WTAU00000000	GCA_013139425.1	96.55	4.08	1	1
WOR-3 bacterium HyVt-237	Guaymas Basin marine hydrothermal sediment	DRBW00000000	GCA_011042705.1	83.52	0.55	1	
WOR-3 bacterium HyVt-388	Guaymas Basin hydrothermal sediment	DRIG00000000	GCA_011052815.1	94.94	1.12	1	1
WOR-3 bacterium JGI_Cruoil_03_44_89	California oil polluted marine water	NOZQ00000000	GCA_002245405.1	95.60	8.39	1	1
WOR-3 bacterium JGI_Cruoil_03_51_56	California oil polluted marine water	NOZP00000000	GCA_002245415.1	93.55	3.71	1	1
WOR-3 bacterium K_DeepCast_150m_m2_040	Tanzania Lake Tanganyik freshwater	VGIR00000000	GCA_016867815.1	94.51	4.40	1	1
WOR-3 bacterium Kmv43	Ukraina active mud volcano bubbling pool	JACUUY000000000	GCA_014859085.1	94.51	1.10	1	1
WOR-3 bacterium NC_groundwater_1521_Pr4_B-0.1um_38_3	California groundwater	JACQXS000000000	GCA_016208615.1	86.92	1.10	1	
WOR-3 bacterium RBG_13_43_14	Rifle background sediment	MEUM00000000	GCA_001771735.1	61.72	0.00	1	
WOR-3 bacterium RBin_337	Australia hypersaline water microbial mat	WJJO00000000	GCA_014729665.1	84.53	0.00	1	
WOR-3 bacterium RS_13_33	South China Sea deep-sea cold-seep sediment	JAGLYS000000000	GCA_023132105.1	91.08	1.10	1	1
WOR-3 bacterium RS_13_48	South China Sea deep-sea cold-seep sediment	JAGLZA010000000	GCA_023131835.1	94.59	1.10	1	1
WOR-3 bacterium RS_15_7	South China Sea deep-sea cold-seep sediment	JAGMBB000000000	GCA_023129935.1	92.94	0.00	1	1
WOR-3 bacterium RS_16_32	South China Sea deep-sea cold-seep sediment	JAGMBU000000000	GCA_023129615.1	95.60	1.10	1	1
WOR-3 bacterium RS_17_14	South China Sea deep-sea cold-seep sediment	JAGMCL010000000	GCA_023129165.1	92.31	0.00	1	1
WOR-3 bacterium RS_17_4	South China Sea deep-sea cold-seep sediment	JAGMDF000000000	GCA_023128825.1	92.86	1.10	1	1
WOR-3 bacterium RS_17_8	South China Sea deep-sea cold-seep sediment	JAGMDO000000000	GCA_023128675.1	93.04	0.00	1	1
WOR-3 bacterium RS_18_8	South China Sea deep-sea cold-seep sediment	JAGMEL000000000	GCA_023128195.1	90.29	4.50	1	1
WOR-3 bacterium S014_22	Pacific ocean deep-sea hydrothermal deposit	WFRC00000000	GCA_015486685.1	91.14	6.66	1	1
WOR-3 bacterium S014_35	Pacific ocean deep-sea hydrothermal deposit	WFRF00000000	GCA_015486655.1	78.25	1.10	1	
WOR-3 bacterium S141_55	Pacific ocean deep-sea hydrothermal deposit	WFXO00000000	GCA_015490555.1	91.51	0.10	1	1
WOR-3 bacterium S141_60	Pacific ocean deep-sea hydrothermal deposit	WFXS00000000	GCA_015490465.1	91.38	0.00	1	1
WOR-3 bacterium S141_80	Pacific ocean deep-sea hydrothermal deposit	WFYF00000000	GCA_015490255.1	77.50	0.15	1	
WOR-3 bacterium S143_31	Pacific ocean deep-sea hydrothermal deposit	WFZT00000000	GCA_015489415.1	92.31	6.59	1	1
WOR-3 bacterium S143_40	Pacific ocean deep-sea hydrothermal deposit	WGAB00000000	GCA_015489295.1	93.41	8.26	1	1
WOR-3 bacterium S143_50	Pacific ocean deep-sea hydrothermal deposit	WGAI00000000	GCA_015489175.1	93.41	1.10	1	1

WOR-3 bacterium S143_60	Pacific ocean deep-sea hydrothermal deposit	WGAN00000000	GCA_015494805.1	82.60	3.45	1	
WOR-3 bacterium S143_66	Pacific Ocean marine hydrothermal vent	WGAQ00000000	GCA_015494735.1	75.01	2.85	1	
WOR-3 bacterium S146_103	Pacific ocean deep-sea hydrothermal deposit	WGDS00000000	GCA_015493155.1	66.29	0.10	1	
WOR-3 bacterium S146_79	Pacific ocean deep-sea hydrothermal deposit	WGFI00000000	GCA_015492295.1	87.07	0.00	1	
WOR-3 bacterium SKYB20	Alkaline hot spring brown biofilm	JANXBB000000000	GCA_025061975.1	91.01	1.12	1	1
WOR-3 bacterium SKYB27	Alkaline hot spring brown biofilm	JANXBH000000000	GCA_025060695.1	94.92	0.00	1	1
WOR-3 bacterium SM23_42 WORSMTZ_101083	North Carolina methane-rich estuary sediment	LJUJ00000000	GCA_001303785.1	93.41	1.10	1	1
WOR-3 bacterium SpSt-1182	California hot spring sediment	DSBX00000000	GCA_011049385.1	76.84	2.20	1	
WOR-3 bacterium UBA1063	Anaerobic digester	DCDE00000000	GCA_002316275.1	94.92	0.00	1	1
WOR-3 bacterium UBA2202	Canada Suncor tailings pond	DCUY00000000	GCA_002327905.1	92.31	0.00	1	1
WOR-3 bacterium UBA2258	Athabasca oil sand wastewater	DDXO00000000	GCA_002347155.1	94.41	1.10	1	1
WOR-3 bacterium UBA3072	California oil polluted marine water	DFBU00000000	GCA_002367385.1	91.29	0.55	1	1
WOR-3 bacterium UBA3073	California oil polluted marine water	DFBT00000000	GCA_002366725.1	85.16	1.65	1	
WOR-3 bacterium UBA3079	California oil polluted marine water	DFBN00000000	GCA_002366635.1	85.31	1.10	1	
WOR-3 bacterium UBA4773	Canada Suncor tailings pond	DHHL00000000	GCA_002403255.1	91.66	1.10	1	1
WOR-3 bacterium UBA5618	McMurray wastewater	DIKM00000000	GCA_002424565.1	71.43	0.00	1	
WOR-3 bacterium UBA5631	Wastewater	DIJZ00000000	GCA_002421425.1	96.61	1.69	1	1
WOR-3 bacterium UBA6260	Medicine Hat wastewater	DJVI00000000	GCA_002441125.1	93.41	0.00	1	1
WOR-3 bacterium UBA7921	Terrestrial	DMCX00000000	GCA_003485365.1	90.05	0.00	1	1
WOR-3 bacterium UBA9909	Terrestrial	DQBP00000000	GCA_003526185.1	95.60	0.00	1	1
WOR-3 bacterium UBA9956	Hydrocarbon	DMZY00000000	GCA_003543575.1	81.06	0.10	1	
WOR-3 bacterium Zod_Metabat.145	Zodletone Spring freshwater sediment	JAFGHJ000000000	GCA_016930175.1	94.04	0.00	1	1
WOR-3 bacterium Zod_Metabat.185	Freshwater hot spring sediment	JAFGIG000000000	GCA_016929715.1	90.11	0.00	1	1
WOR-3 bacterium Zod_Metabat.631	Zodletone Spring freshwater sediment	JAFGON000000000	GCA_016926475.1	90.29	0.00	1	1
WOR-3 bacterium Zod_Metabat.699	Freshwater hot spring sediment	JAFGPO000000000	GCA_016936155.1	92.00	2.25	1	1
WOR-3 bacterium Zod_Metabat.732	Freshwater hot spring sediment	JAFGQF000000000	GCA_016935775.1	82.60	1.65	1	
WOR-3 bacterium Zod_Metabat.816	Freshwater hot spring sediment	JAFGRF000000000	GCA_016935295.1	76.86	0.00	1	
WOR-3 bacterium Zod_Metabat.887	Zodletone Spring freshwater sediment	JAFGSK000000000	GCA_016934635.1	95.51	0.00	1	1
WOR-3 bacterium Zod_Metabat.912	Freshwater hot spring sediment	JAFGSQ000000000	GCA_016934535.1	94.14	3.30	1	1

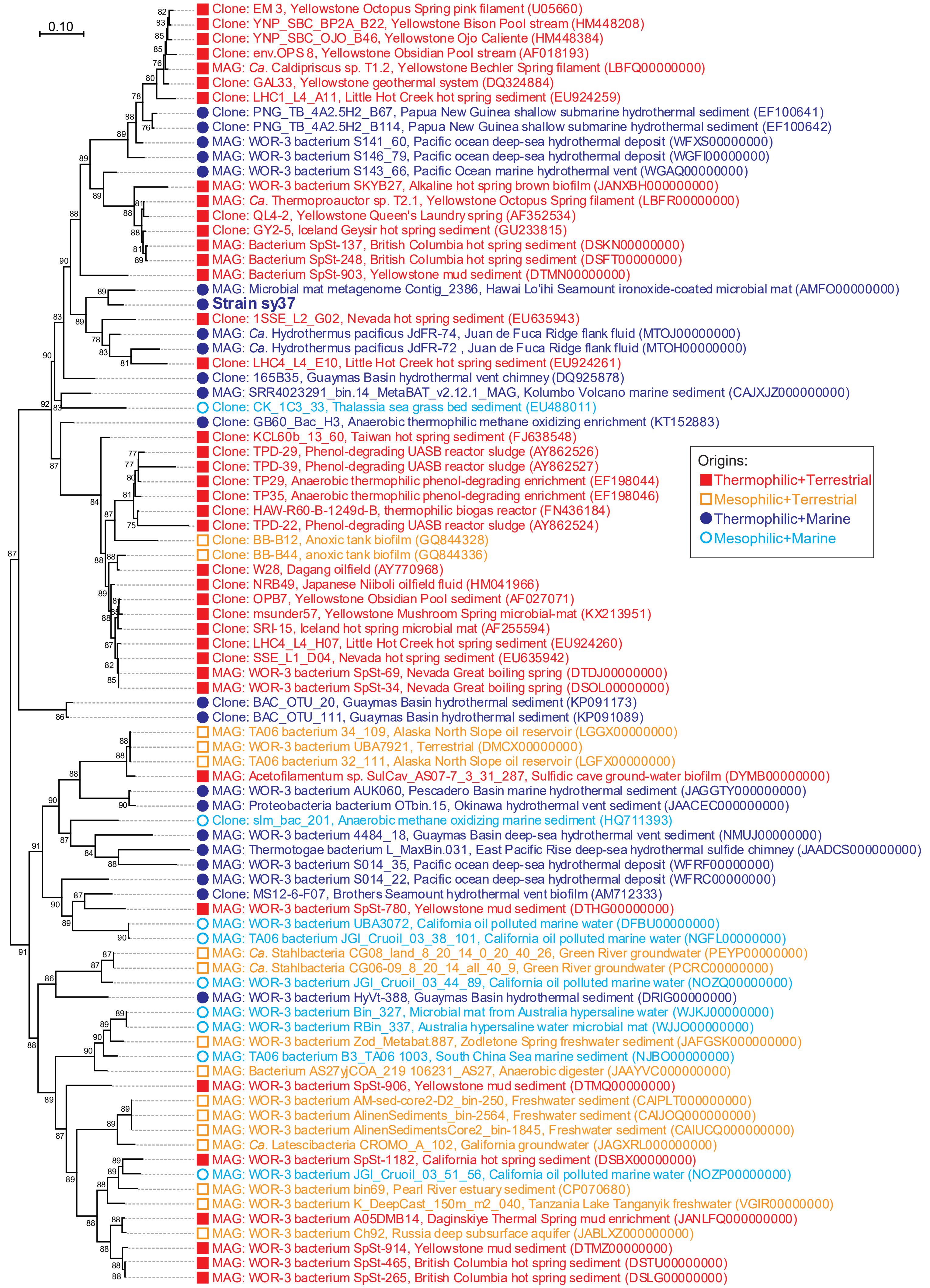

Fig. S1. Maximum-likelihood tree of strain sy37 within the WOR-3 lineage based on 16S rRNA gene sequences. Solid squares (Red), open squares (orange), solid circles (dark blue), and open circles (blue) indicate origins from thermophilic terrestrial, mesophilic terrestrial, thermophilic marine, and mesophilic marine environments, respectively.

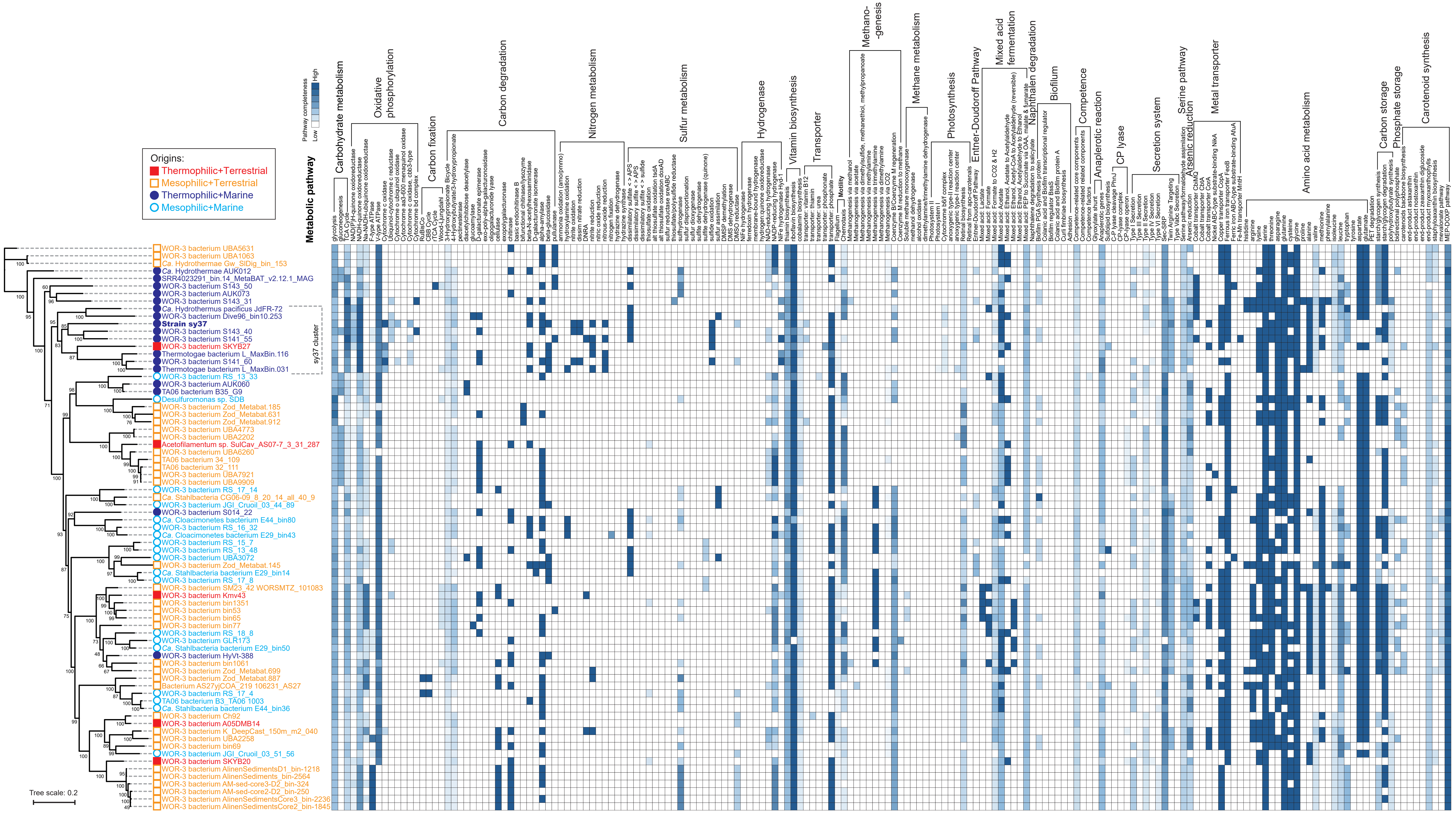

Fig. S2. Phylogenetic tree among strain sy37 and the authentic MAGs of WOR-3 lineage, and the heat map of their metabolic pathway completeness inferred from PhyoPhlAn and KEGG-Decoder, respectively. The accession numbers used for the analysis are shown in supplemental Table S1. Bootstrap probabilities are indicated at branching points. Solid squares (Red), open squares (orange), solid circles (dark blue), and open circles (blue) indicate origins from thermophilic terrestrial, mesophilic terrestrial, thermophilic marine, and mesophilic marine environments, respectively. In the map, dark blue colors represent complete or highly complete pathways and lighter colors do non or highly incomplete pathways. Bar, 0.2 substitutions per amino acid site.
